# Back Pain: An Ominous Harbinger of Aortitis

**DOI:** 10.7759/cureus.3711

**Published:** 2018-12-11

**Authors:** Saurav Suman, Amulya Prakash, Shista Priyadarshini, Prerna Dogra

**Affiliations:** 1 Internal Medicine, University of Kentucky, Lexington, USA; 2 Internal Medicine, Monmouth Medical Center, Long Branch, USA

**Keywords:** aortitis, vasculitis

## Abstract

Isolated aortitis is a rare entity and was recently included in the 2012 Revised International Chapel Hill Consensus Conference Nomenclature of Vasculitides under the single organ vasculitis group. Isolated aortitis represents a challenging situation due to the lack of reliable diagnostic methodology. Here, we describe the case of a 46-year-old woman who presented with severe upper back pain. She tested negative for pulmonary embolism, myocardial infarction, and other usual causes of back pain. The case highlights the clinical presentation of a rare disease with a usual symptom.

## Introduction

The diagnosis of vasculitic disorders offers challenges for the clinician. We report a case of single organ vasculitis involving the aorta, which presented as chest and back pain that posed significant diagnostic challenges despite excellent diagnostic tools.

## Case presentation

**Figure 1:**
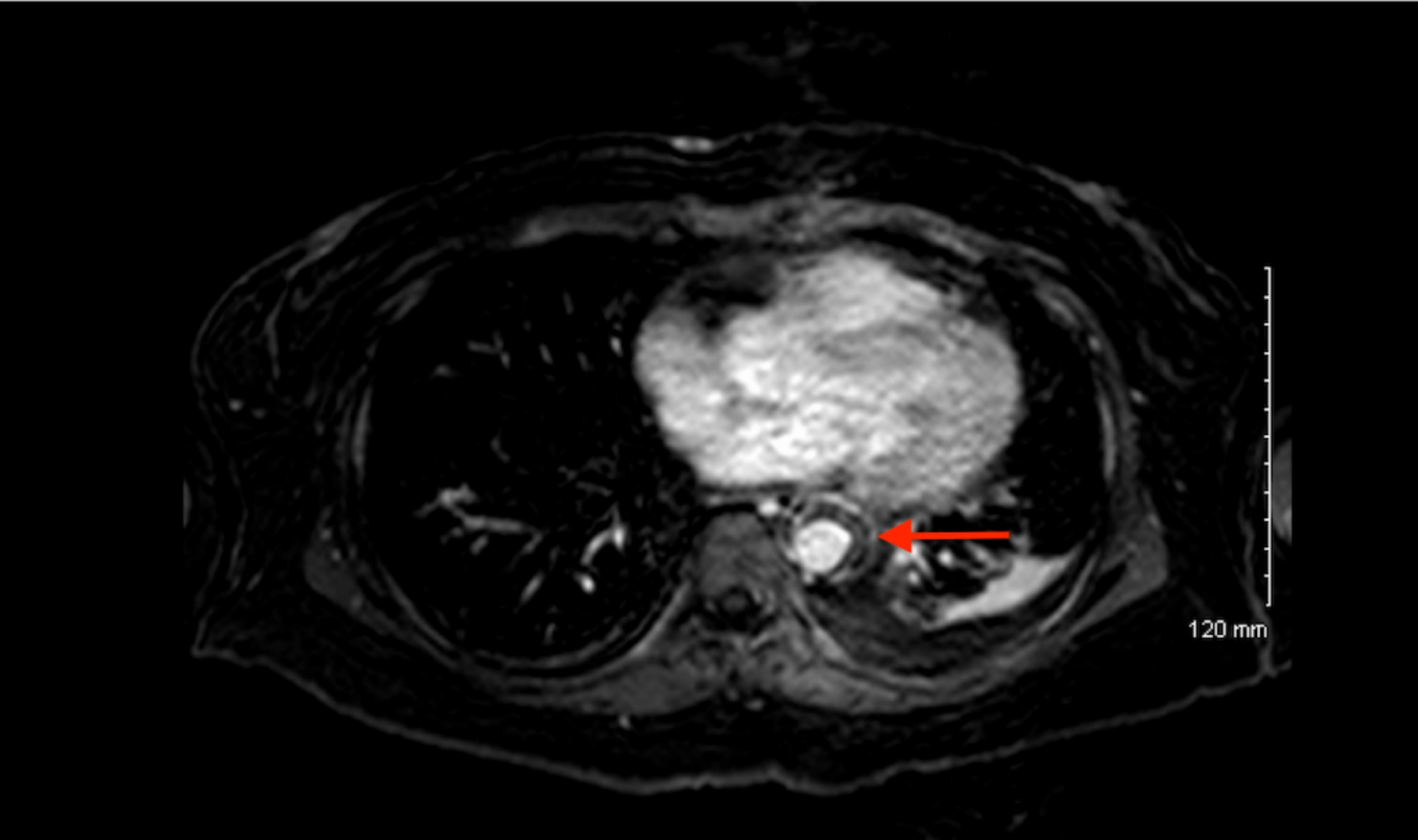
MRI of the aortic wall The red arrow indicates necrosis of the aortic wall. Notice the enhancement of the outer and inner layers of the aortic wall and non-enhancing middle layer. MRI: magnetic resonance imaging

**Figure 2:**
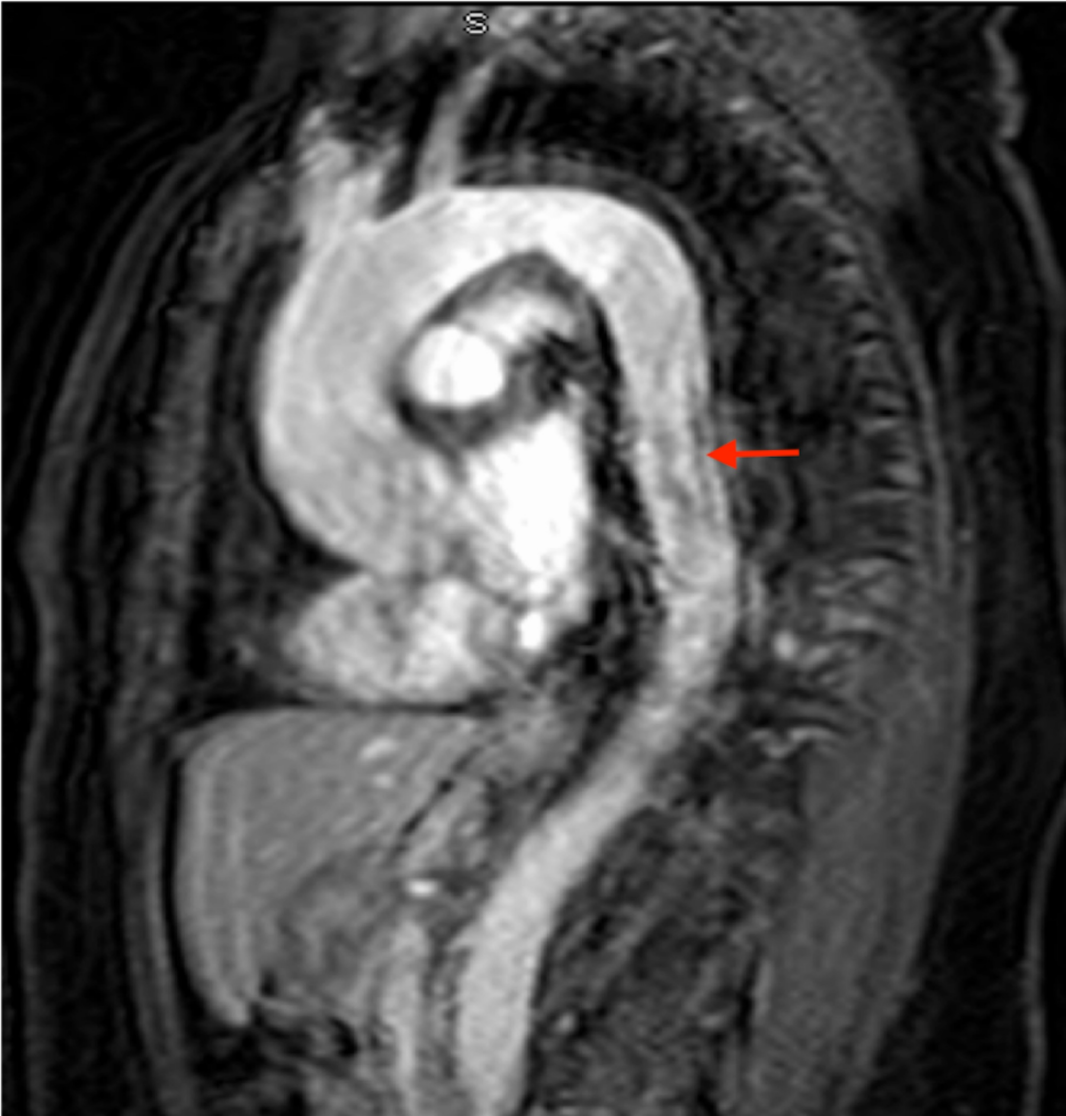
Coronal MRI of the aortic wall The red arrow indicates necrosis in the aortic wall due to aortitis. MRI: magnetic resonance imaging

**Figure 3:**
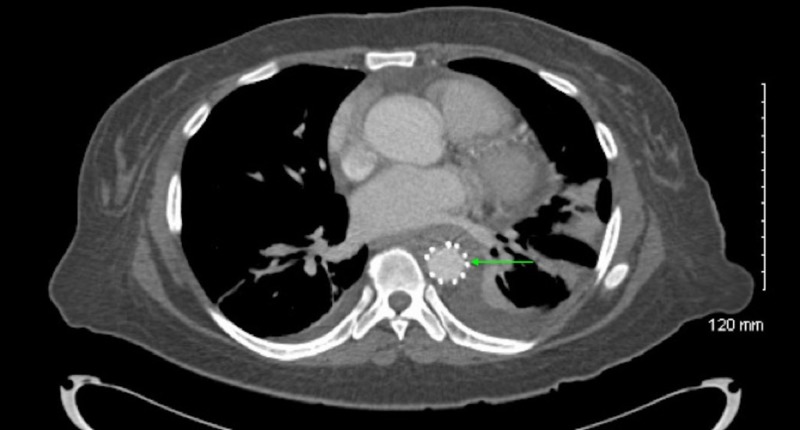
CT scan of chest post-procedure Green arrow showing TEVAR aortic repair CT: computed tomography; TEVAR: thoracic endovascular aortic repair

This is the case of a 46-year-old African American woman with a history of hypertension, who presented with upper back pain that began a week prior to presentation. The pain was intermittent, sharp, 8/10, radiated to the lower back, aggravated by movement, especially on leaning forward; and not relieved by nonsteroidal anti-inflammatory drugs (NSAIDs). The patient denied any weakness, numbness, bowel, or urinary incontinence. The morning of the presentation, the patient was awoken from her sleep by 10/10 mid-sternal chest pain. Pain was pressure-like in character with no associated symptoms. Initial emergency room (ER) vitals revealed a difference in blood pressure of approximately 20 mmHg between the lower and upper extremities (right arm, 170/115; left arm 179/112; right leg 202/115; left leg 197/126) and the chest pain disappeared with narcotics; however, the intermittent upper back pain continued. The physical examination was significant for reproducible midsternal chest pain. Initial labs were significant for very high acute phase reactants, marginally elevated D-dimer at 1.96, and negative troponins. Computed tomography (CT) angiogram ruled out pulmonary embolism (PE) but revealed circumferential thickening of the descending aorta and a mildly ectatic ascending aorta. Subsequently, aortic magnetic resonance imaging (MRI) with contrast showed an enhancement of adventitia with non-enhancing media (Figures 1-2). This was thought to be either due to aortitis or aortic dissection. Subsequent trans-esophageal echocardiography was not consistent with aortic dissection, making aortitis the most probable diagnosis. Infectious tests with blood culture were unremarkable. Additionally, tests for HIV, viral hepatitis, and syphilis were also negative. Vasculitis tests, such as antinuclear antibody (ANA), antineutrophil cytoplasmic antibody (ANCA), and anti-cyclic citrullinated peptide (anti-CCP), were also normal. The patient was diagnosed with single organ (aorta) vasculitis and was started on high-dose corticosteroid. Due to the persistence of severe chest pain, a decision was made to proceed with thoracic endovascular aortic repair (TEVAR; Figure 3). Immediately post-procedure, the patient experienced a dramatic improvement in pain and was discharged on a slow, tapering course of corticosteroid.

## Discussion

Aortitis means the inflammation of the wall of the aorta [1]. It could be a manifestation of large vessel vasculitis, such as giant cell arteritis (GCA) and temporal arteritis (TA), or could be related to other rheumatological conditions, such as rheumatoid arthritis or systemic lupus erythematosus (SLE) [1]. Isolated large vessel aortitis has also been described, idiopathic vs. immunoglobulin G4 (IgG4) related disease [2]. Additionally, aortitis could be secondary to microbial infections as well, such as Staphylococcus aureus, Streptococcus pneumonia, Salmonella, Treponema pallidum (syphilis), Mycobacterium tuberculosis, etc. [1]. The pathogenesis of microbial-induced aortitis mainly involves the adherence of the microbe to atherosclerotic plaque in the vessel, causing inflammation and destruction of the aortic wall [3].

In an attempt to classify aortitis, Burke et al., in 2008, studied the pathology of 52 aortic specimens and proposed a histopathological classification of aortitis [4]. However, they excluded the infectious etiology of aortitis from their study. Therefore, they classified the non-infectious variant of aortitis. Their study involved examining all the specimens under a microscope for necrosis, inflammation, regenerative changes, and fibrosis in the aortic wall. The net result was a classification of non-infectious aortitis as necrotizing aortitis (NA) vs. non-necrotizing aortitis (NNA).

Necrotizing aortitis involves laminar necrosis of the media with giant cell reaction and destruction of the elastic fibers [5]. Over time, the media heals with relatively less fibrosis but with extensive fibrosis of the adventitia and intimal layers. This extensive fibrosis leads to an increase in wall thickening. On imaging, it appears thick-walled and is more likely to have symptoms of obstruction; however, aneurysms can also be found [2]. On the other hand, non-necrotizing aortitis does not result in medial necrosis or elastic loss; however, inflammatory cells are present in the media. It more commonly presents as an aortic aneurysm and aortic dissection. NNA is exclusively present in the elderly and a majority of these patients have a prior history of temporal arteritis (TA) [4]. On the contrary, NA has a bimodal age distribution (1. Adults of age < 65 yrs; and 2. Elderly of age > 65 yrs), and the adult form shares a histologic similarity with Takayasu arteritis [5]. In general, distinguishing between GCA and Takayasu arteritis can be challenging as they have very similar clinical and histopathological findings. One of the unique features of 4GCA is the presence of “skip lesions,” which can lead to a false negative finding on temporal artery biopsy [6]. Takayasu arteritis can be diagnosed with the help of diagnostic criteria that were first developed by Ishikawal et. al in 1988 and later modified by Sharma et al. in 1995 [7-9].

IgG4-related disease is a relatively new discovery that has been found to affect multiple organ systems [2]. There have been several case reports attributing IgG4-related disease as causing single vessel vasculitis [2,10]. The diagnosis of IgG4-related disease is mainly based on the combination of clinical features and the characteristic histopathological finding [9].

The detection of aortitis requires advanced imaging, such as a CT scan or MRI. Aortitis can be identified on CT angiography (CTA) and the walls of the aorta can be better studied by MRI. CT can demonstrate the wall thickening and the periaortic inflammation, but it is not very sensitive to detect mild inflammation and wall edema [11]. However, CT is an excellent modality to screen patients for recurrent aortitis after being treated [1]. MRI provides a superior imaging technique as compared to CT and is able to detect the vessel edema using the edema-weighted technique [12]. 18-Fluorodeoxyglucose (18-FDG) positron-emission tomography (PET) scanning in combination with MRI (hybrid imaging) is gaining popularity to correctly diagnose cases of aortitis with better accuracy [13]. Transesophageal echocardiogram (TEE) can provide valuable information in the evaluation of aortitis of the thoracic aorta. It can correctly measure the degree of aortic regurgitation, aneurysmal dilatation, and dissection [14].

The approach to management depends on the underlying cause. The aim of therapy includes both immediate treatment of aortic inflammation, infection, if any, and surveillance and management of aortic and arterial complications.

Infectious aortitis

Although uncommon, it requires rapid diagnosis with the initiation of antibiotic therapy. The initial treatment is intravenous antibiotics with broad antimicrobial coverage, particularly against Staphylococcus species and gram-negative rods. It should be started as soon as the diagnosis is suspected and should be tapered based on culture and sensitivity data, for six to 12 weeks after the clearance of blood cultures [15]. Due to the high rate of mortality among patients with a gram-negative infection of aortitis, a combination strategy of intensive antibiotic therapy and surgical debridement with aneurysm repair is suggested.

Aortitis associated with large vessel vasculitis

Immunosuppressive therapy is the mainstay of treatment. In general, a starting dose of prednisolone 40-60 mg per day for GCA and 1 mg/kg/day for TA is suggested [15]. The prednisolone dose is gradually tapered over time with dose monitoring for symptoms and vascular signs, inflammatory markers, and imaging studies. Despite glucocorticoid therapy, the relapse rate for both GCA and TA are as high as 50% [1,16-17]. Due to the need for long-term therapy and the potential adverse effects, medication to prevent osteoporosis, gastric ulcerations, Pneumocystis jiroveci pneumonia should also be prescribed and monitored for the development of steroid-induced diabetes mellitus, hypertension, cataract, and secondary infections.

Isolated idiopathic aortitis

The optimal management of such patients is uncertain, and the decision to treat with a course of glucocorticoid therapy should be considered depending upon the presentation, site, and extent of the inflammation. Patients with isolated idiopathic aortitis require careful follow-up, as they have a propensity toward aneurysm formation in other vascular beds over time.

## Conclusions

The symptoms of aortitis present as vague chest and back pain,and early diagnosis can easily be missed, as advanced imaging, such as CT scans or MRI, is required to diagnose the condition. Keeping this differential in mind is important in relevant cases. A delayed or missed diagnosis will have high mortality or long-term complications.
